# Comparison of the Efficacy and Safety of Aspirin and Rivaroxaban Following Enoxaparin Treatment for Prevention of Venous Thromboembolism after Hip Fracture Surgery

**DOI:** 10.1111/os.12542

**Published:** 2019-10-29

**Authors:** Qiang Huang, Shu‐xing Xing, Yi Zeng, Hai‐bo Si, Zong‐ke Zhou, Bin Shen

**Affiliations:** ^1^ Department of Orthopedic Surgery West China Hospital, Sichuan University Chengdu Sichuan Province China; ^2^ Department of Orthopedic Surgery Chengdu Fifth People's Hospital Chengdu Sichuan Province China

**Keywords:** Aspirin, Prophylaxis, Hip Fracture, Rivaroxaban, Venous Thromboembolism

## Abstract

**Objective:**

To compare the efficacy and safety of aspirin with rivaroxaban following treatment with enoxaparin for prevention of venous thromboembolism (VTE) after hip fracture surgery (HFS).

**Methods:**

A total of 390 patients were enrolled in the trial. According to an odd or even number at the end of their registration number, the patients were divided into the aspirin group (n = 198) and the rivaroxaban group (n = 192). All patients were given enoxaparin subcutaneous injection after the operation and returned to the routine dose the next day until postoperative day five. The patients in the aspirin group received an additional 16 days of thromboprophylaxis with 100 mg of aspirin once daily. The rivaroxaban group was assigned to receive an additional 16 days of thromboprophylaxis with 10 mg of oral rivaroxaban once daily. Patients were followed for 90 days regarding VTE and bleeding complications.

**Results:**

The incidence of VTE in the aspirin group and rivaroxaban group was 6.6% (13/198) and 5.7% (11/192), respectively (*P =* 0.83). The rate of major bleeding events occurred in two (1.0%) patients in the aspirin group and in one patient (0.5%) in the rivaroxaban group (*P* = 1.0). A combination of major bleeding and clinically relevant nonmajor bleeding occurred in five patients (2.5%) in the aspirin group and in six patients (3.1%) in the rivaroxaban group (*P* = 0.77). During the 90‐day follow‐up, a pulmonary embolism developed in one patient (0.5%) in the aspirin group and none in the rivaroxaban group (*P* = 1.0).

**Conclusions:**

Extended prophylaxis for 21 days with aspirin was equivalent to the direct oral anticoagulant rivaroxaban after hip fracture surgery with an initial 5‐day postoperative course of enoxaparin. Aspirin may be an effective, safe, convenient, and cheap alternative for extended prophylaxis after hip fracture surgery.

## Introduction

Venous thromboembolism (VTE) is a serious complication with a high incidence during and after hospitalization, and it is also an important factor in perioperative mortality and unexpected deaths in hospitals^1^, ^2^, ^3^, ^4^, ^5^, ^6^. An epidemiological study demonstrated that the incidence of deep vein thrombosis (DVT) after THA, TKA, and hip fracture surgery (HFS) without pharmacological thromboprophylaxis at 19 orthopedic centers in seven Asian countries was 41%^7^. Total DVT and proximal DVT rates were highest in total knee arthroplasty (TKA) patients (58.1% and 17.1%), followed by HFS patients (42.0% and 7.2%). In China, the incidence of DVT without prevention after total hip arthroplasty (THA) was 20.6%–47.1%, and TKA was 30.8%–58.2% [4]. The incidence of proximal DVT without preventive measures after HFS was 15.7%^4^, ^8^. Previous studies have reported that, without any prophylaxis, pulmonary embolism (PE) is responsible for 5%–10% of inpatient deaths; the incidence of PE inpatient fatalities was found to be 2%–3% after elective hip arthroplasty and 4%–7% after hip fracture surgery^9^, ^10^, ^11^, ^12^, ^13^.

After major orthopaedic surgery (TKA, THA, HFS), the increased coagulability of blood may persist for up to 4 weeks, while the increased risk of postoperative DVT may last for 3 months. Most episodes of VTE occur in discharged patients and have led some authors to evaluate the efficiency of extended prophylaxis after major orthopaedic surgery^14^, ^15^, ^16^, ^17^, ^18^, ^19^. In patients who underwent HFS, it is recommended that the duration of drug prevention be at least 10 days and extendable to 11–35 days^3^, ^4^, ^20^. Several studies have shown that extended prophylaxis substantially reduces the risk of VTE, and they recommend a longer prophylaxis duration in all patients undergoing major orthopaedic surgery^14^, ^15^, ^17^, ^21^, ^22^, ^23^, ^24^. Due to the high risk of VTE after HFS, the UK‐based National Institute for Health and Clinical Excellence (NICE, 2018), Scottish Intercollegiate Guidelines Network (SIGN, 2009), the American College of Clinical Pharmacy (ACCP, 2012) and the American Academy of Orthopaedic Surgeons (AAOS, 2011) guidelines suggest that the use of low‐molecular‐weight heparin (LMWH) is preferable to other agents for prevention of thrombosis^25^, ^26^, ^27^, ^28^.

In patients undergoing major orthopaedic surgery, the guidelines recommend the use of one of the following for prophylaxis: LMWH, fondaparinux inhibiting activated factor Xa (rivaroxaban, apixaban, edoxaban), and aspirin (Grade 1B) for a minimum of 10–14 days^26^. The weight‐based dosing of enoxaparin is also effective in preventing VTE, a kind of LMWH^29^, ^30^. In comparison to factor Xa inhibitors, enoxaparin is associated with higher rates of postoperative anemia. According to European guidelines, perioperative thromboprophylaxis is essential in elderly patients, for whom direct oral anti‐coagulants (Grade 1C) are effective and well‐tolerated^31^. Although rivaroxaban has the same thromboprophylaxis effect as LMWH, it is accompanied by a higher risk of hemorrhagic complications and wound complications^32^, ^33^, ^34^, ^35^, ^36^, ^37^.

The 2007 AAOS guidelines recommend aspirin for patients at higher risk of bleeding events (Grade 3C), because aspirin may be associated with less bleeding after major orthopaedic surgery than other pharmacological agents^38^, ^39^. The 2012 ACCP guidelines recommend the use of aspirin as VTE prophylaxis for patients undergoing THR, TKR, or HFS (Grade 1B)^26^. Inexpensive prophylaxis could be suitable for low‐income countries, but adequate large‐scale trials with proper study designs should be conducted^39^.

A randomized trial has shown that extended prophylaxis for 28 days with aspirin was noninferior to and as safe as dalteparin for the prevention of VTE after THA^40^. Anderson *et al*.^15^ performed a multicenter, double‐blind, randomized, controlled trial and found that extended prophylaxis with aspirin was not significantly different from rivaroxaban after hip or knee arthroplasty. The above clinical trials have suggested that aspirin may be effective for extended prevention of VTE after THA or TKA. We searched PubMed/Medline, CENTRAL, Embase, and Web of Science using the terms *aspirin, rivaroxaban, low‐molecular‐weight heparin, hip fracture, deep vein thrombosis* for studies of extended prophylaxis comparing aspirin to rivaroxaban after surgery for hip fractures and found a lack of reports.

The purpose of this study was to: (i) compare the incidence of VTE with aspirin and rivaroxaban for the prevention of VTE after hip fracture surgery;(ii) study the incidence of bleeding events with aspirin and rivaroxaban;(iii) determine whether the extended prophylaxis anticoagulation scheme was reasonable.

## Materials and methods

### *Study Population*

The present study retrospectively analyzed 581 patients with hip fracture who were admitted to the Orthopedics Department of Chengdu Fifth People's Hospital from November 2011 to March 2018.

### *Inclusion and Exclusion Criteria*

The inclusion criteria in our study were: (i) patients who were diagnosed with hip fracture by X‐ray and/or CT and underwent hip fracture surgery were invited to participate in our study; (ii) all patients received enoxaparin (Sanofi company, Paris, France) subcutaneously for 5 days after hip fracture surgery, and aspirin (Bayer Pharma) or rivaroxaban (Xarelto, Bayer Pharma) orally prevented VTE until 21 days after operation; (iii) after the 90‐day follow‐up, we compared the primary effectiveness outcomes (VTE, PE) and safety outcomes (bleeding events) of the two groups; and (iv) patients were requested to agree to a random selection of the use of aspirin or rivaroxaban for anticoagulation.

The exclusion criteria were as follows: (i) lower extremity DVT confirmed by preoperative ultrasonography; (ii) a history of thromboembolic disease and undergoing anticoagulant therapy; (iii) the presence of hemorrhagic diseases and/or a major bleeding history; (iv) severe liver or kidney diseases; (v) coagulation disorders; (vi) an allergy to enoxaparin, aspirin, or rivaroxaban; and (vii) a platelet count less than 100 * 10^9^ cells/L. The present study was approved by the Medical Ethics Committee of Chengdu Fifth People's Hospital. At beginning of the study, the subjects were informed of the purpose, potential benefits, and risks of the trial. All subjects were voluntary participants who provided informed consent. There was no industry support or funding.

**Figure 1:**
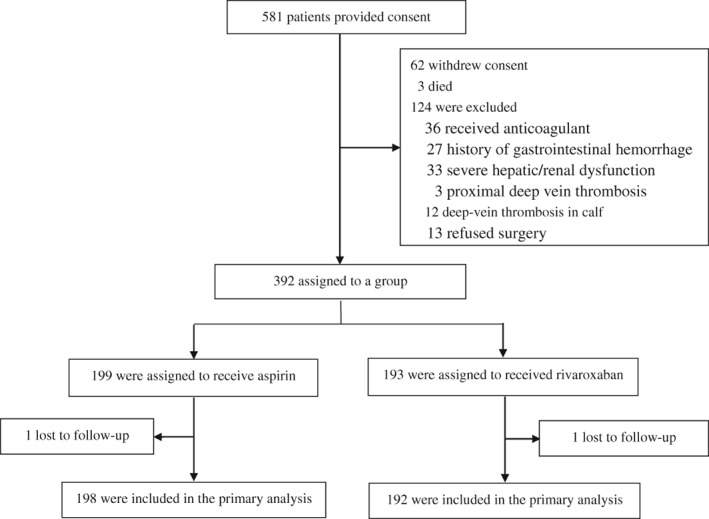
Enrollment and outcomes.

A total of 581 patients who had suffered hip fracture consented to participate in the study; 191 patients were excluded according to the exclusion criteria (Fig. 1). Before the operation, 36 patients received anticoagulant treatment because of cardiovascular disease, 27 patients had a history of gastrointestinal hemorrhage, 33 patients had severe hepatic/renal dysfunction, and 13 patients refused surgery. DVT was found in 15 patients before the operation, including three patients with proximal DVT who received drug anticoagulation therapy. Of the remaining 457 patients, two died of heart failure (<10 days after hip fracture), one died of a lung infection before the operation (15 days after hip fracture), 27 withdrew their consent before the operation, and an additional 35 patients were excluded during the postoperative period before grouping. The three patients who died before surgery were due to severe comorbidities, which represented early deaths according to the Rosso *et al*.^41^ and Castronuovo *et al*.^42^ elderly hip fracture death staging method: early (30 days), mid (6 months), and late (1 year). The remaining 392 patients were assigned to a group. Two patients were lost during follow‐up and were not included in any of the analyses. No other patients were lost to follow‐up. A total of 249 patients underwent intertrochanteric fractures of the femur, and 141 underwent femoral neck fractures. According to an odd or even number at the end of their registration number, 390 patients were divided into the aspirin group (n = 198) and the rivaroxaban group (n = 192).

### *Treatment Protocol*

#### *Preoperative Treatment*

All patients were preoperatively treated with equal‐length contraction of quadriceps muscle and flexion and extension of the ankle joint combined with an intermittent pneumatic compression device to prevent DVT on the day of admission^26^. Ultrasound examination of lower limb veins is a routine examination, including deep femoral vein, superficial femoral vein, popliteal vein, anterior tibial vein, posterior tibial vein, and peroneal vein. All patients had to be examined before and after surgery. DVT was diagnosed according to the criteria of Fraser *et al*.^43^. Skin traction of the lower extremity was conducted for all patients following admission.

#### *Surgical Approach*

The surgeries were carried out by the same team of doctors. According to the patient's situation, epidural anesthesia or general anesthesia was administered. According to the femoral neck fracture classification of Garden,^44^ type 1 and type 2 fractures were supine on the operating table, then traction reduction was performed and internally fixed with three hollow screws through direct lateral approach. Displaced subcapital (Garden 3 and 4) hip fractures were excised femoral head, and treated with uncemented hemiarthroplasty through lateral decubitus position and posterolateral approach. Intertrochanteric and subtrochanteric fractures were supine on the operating table, treated with closed or open reduction followed by internal fixation with a sliding hip screw or proximal femoral nail anti‐rotation (PFNA) through direct lateral approach. Intravenous second‐generation cephalosporins were administered 30 min before surgery as prophylaxis for infection. Wound drainage was removed within the first postoperative 48 h, and the amount of discharge was recorded, if the drainage tube was inserted intraoperatively.

#### *Anticoagulation Scheme*

All patients were given a subcutaneous injection of enoxaparin 2000 U 4–6 h after the operation (2–4 h after the removal of the epidural catheter) and returned to the routine dose the next day. This regimen was followed by daily administration up to and including day five after surgery. The patients in the aspirin group received an additional 16 days of thromboprophylaxis with 100 mg of aspirin once daily, starting on day six after surgery. The rivaroxaban group was then assigned to receive an additional 16 days of thromboprophylaxis with 10 mg of oral rivaroxaban once daily, starting on postoperative day six. The anticoagulation duration used in this study was 21 days because a previous study report with extended prophylaxis for 3 weeks after hip fracture surgery showed a reduced risk of VTE by 96% and was well tolerated^18^. Early mobilization was encouraged with weight bearing as tolerated, according to European guidelines on perioperative VTE prophylaxis^31^.

The time to surgery was calculated as the time between when the fracture was reported by the patient and the time the operation was performed. Intraoperative blood loss was calculated by measuring the amount of fluid in the collection containers together with the net swab weight and subtracting the amount of fluid used for lavage. Measured blood loss was calculated as the sum of the intraoperative blood loss and drainage discharge. Blood transfusions were given when hemoglobin levels dropped below 71 g/L or when patients were symptomatic.

### *Outcome Measure*

#### *Venous Thromboembolism*

The primary effectiveness outcome was adjudicated VTE, which was defined as abnormal coagulation of venous blood, resulting in complete or incomplete vascular obstruction. It includes two different clinical manifestations which occur at different stages and in different parts of the body: DVT and pulmonary embolism (PE). After major orthopaedic surgery, VTE is one of the main causes of death of patients in the perioperative period and is also an important cause of unexpected hospital deaths. DVT was diagnosed according to the criteria of Fraser *et al*.^43^. In addition to routine lower extremity venous ultrasound, compression venous ultrasonography was used for suspected patients. The diagnosis of lower extremity venous thrombosis by probe compression observation, and the fact that the vein cannot be compressed or there is no blood flow signal in the venous cava is a specific feature of DVT. The sensitivity and specificity of compression venous ultrasonography in the diagnosis of proximal thrombosis were 90% and 95%.

### *Pulmonary Embolism*

PE refers to disorders of pulmonary circulation and respiratory function caused by thrombi from the venous system or right heart obstructing the pulmonary artery or its branches. PE is one of the important causes of orthopaedic perioperative death. PE was confirmed by computed tomographic pulmonary angiography, according to Goldhaber's^45^ criteria: (i) Symptoms and signs: dyspnea, chest pain, syncope, hemoptysis, etc.; (ii) Electrocardiogram: an S wave in lead I, Q wave in lead III, and T‐wave inversion in leads III, AVF, and V1–V4, incomplete right bundle branch block; (iii) Blood gas analysis: hypoxemia, hypocarbonemia, alveolar and arterial oxygen partial pressure difference increased; (iv) D‐dimer > 500 ug/L; (v) Echocardiography: enlargement of right atrium and right ventricle, weakening of right ventricular motion, and pulmonary hypertension; (vi) Radionuclide lung scan: perfusion defect with pulmonary segment distribution does not match ventilation imaging; (vii) Thoracic spiral CT: low density filling defect or complete filling defect in pulmonary artery; and (viii) Pulmonary angiography: filling defect of pulmonary artery, blocking of blood flow with or without orbital sign.

### *Deep‐vein Thrombosis in the Calf*

Lower limb DVT can be divided as proximal and distal (calf veins)^1^, ^46^. The diameters of the proximal venous vessels are large, which are prone to fatal PTE when the thrombus is released. DVT of the calf veins due to a small diameter was not analyzed as a primary outcome of the trial. When proximal DVT was diagnosed, the prophylactic anticoagulant regimen was changed to therapeutic anticoagulation.

### *Bleeding Events*

The primary safety outcome was bleeding events, including major, clinically relevant bleeding, and minor bleeding according to Anderson's criteria^40^. The primary safety endpoint was bleeding, which was described as major if it was overt and fulfilled at least one of the following criteria: fatal bleeding, symptomatic bleeding into a critical area or organ, or bleeding that caused a 20g/L decrease or more in hemoglobin level or led to transfusion of two or more units of whole blood or red blood cells. Clinically relevant bleeding was defined as clinically relevant but nonmajor if it resulted in hospitalization, reoperation, aspiration, or a wound hematoma complicated by infection. Minor bleeding was defined as overt bleeding that did not fall into one of the aforementioned categories.

Secondary outcome measures were death, myocardial infarction, stroke, and wound infection. Patients with symptoms of PE were evaluated with computed tomographic pulmonary angiography, which is not used as routine screening. Patients who were excluded by computed tomographic pulmonary angiography could continue to receive the trial anticoagulant, but they did not receive additional anticoagulant therapy and were follow‐up over the 90‐day period after the grouping.

### *Statistical Analysis*

The estimated baseline rate of VTE in the rivaroxaban group was 6%, according to the reported incidence of deep venous thrombosis after major orthopaedic surgery in China^47^. A minimal, clinically important difference was established to be 13 percentage point on the basis of literature^32^. Using an equivalence test design we calculated that a sample size of 155 patients per group would provide a power of 90% to show that aspirin was equal to rivaroxaban for the prevention of the primary effectiveness outcome. To account for withdrawal of consent or loss to follow‐up over the course of the trial, we increased the final sample size to 390.

All data were processed using the SPSS19.0 statistical package (IBM, USA). Continuous data are presented as the mean ± standard deviation and analyzed by the independent samples t‐test. Categorical data were expressed as N (%), and the chi‐square test was used; a *P*‐value less than 0.05 (*P* < 0.05) was considered statistically significant.

## Results

### *Baseline Characteristics of Patients in Two Groups*

The mean age of the patients was 68.7 ± 17.1 years, and 48.7% were men. No statistically significant differences were observed in the baseline clinical data, namely, age, gender, fracture site, and combined diseases including type 2 diabetes, chronic obstructive pulmonary disease (COPD), coronary heart disease, and hypertension. There was no significant difference in surgery waiting time, estimated blood loss, time in operation room, and length of hospital stay between the two groups. The characteristics of the patients are shown in Table 1.

**Table 1:** Characteristics of the patients at baseline

Characteristics	Aspirin (n = 198)	Rivaroxaban (n = 192)	*P*‐value
Age ‐ yr.	69.4 ± 17.4	67.8 ± 16.9	0.96
Male ‐ no. (%)	99 (50.0)	91(47.7)	0.61
Body mass index (kg/m^2^)	23.5 ± 2.4	23.7 ± 2.8	0.08
Fracture site			
Femoral neck fracture ‐ no. (%)	72(36.3)	69(35.9)	0.93
Intertrochanteric fracture of the femur ‐ no. (%)	126(63.6)	123(64.1)	
Surgery waiting time ‐ d	4.8 ± 1.7	5.5 ± 1.8	0.50
Type of surgery			
Three hollow screws ‐ no. (%)	23(11.6)	27(14.1)	0.67
PFNA or sliding hip screws ‐no. (%)	126(63.6)	123(64.1)	
Hemi‐arthroplasty ‐ no. (%)	49(24.7)	42(21.9)	
Time in operation room ‐ hr.	1.36 ± 0.33	1.40 ± 0.34	0.73
Estimated blood loss ‐ ml	98.5 ± 60.6	101.2 ± 57.5	0.94
Combined disease			
Type 2 diabetes ‐ no. (%)	17(8.6)	11(5.7)	0.50
COPD ‐ no. (%)	12(6.1)	13(6.8)	
Coronary heart disease ‐ no. (%)	2(1)	0	
Hypertension ‐ no. (%)	24(12.1)	22(11.5)	
Length of hospital stay ‐ d	21.7 ± 12.5	19.8 ± 8.1	0.09

### *Postoperative VTE*

The incidence of VTE in the aspirin and rivaroxaban group was 6.6% (13/198) and 5.7% (11/192) respectively (*P* = 0.83, Table 2). During the 90‐day follow‐up, a PE developed in one patient (0.5%) in the aspirin group and none in the rivaroxaban group (*P* = 1.0, Table 2). Each of the two groups consisted of no cases of lethal PE. DVT of the calf occurred in 21 patients(10.6%) in the aspirin group and in 19 patients(9.9%) in the rivaroxaban group (*P* = 0.87, Table 2).

**Table 2:** Comparison of the incidence of postoperative VTE between the two groups

Outcomes	Aspirin (n = 198)	Rivaroxaban (n = 192)	*P‐*value
	*no.%*	*no.%*	
Venous thromboembolism	13(6.6)	11(5.7)	0.83
Pulmonary embolism	1 (0.5)	0 (0)	1.00
Proximal DVT	12 (6.1)	10 (5.2)	0.83
Pulmonary embolism + Proximal DVT	0	1(0.5)	0.49
DVT in the calf	21 (10.6)	19(9.9)	0.87

*Note*: No lethal pulmonary embolism occurred in both groups.

### *Bleeding Outcomes*

Major bleeding events in the aspirin and rivaroxaban group occurred in two patients (1.0%) and one patient (0.5%), respectively (*P* = 1.0, Table 3). In the aspirin group, one patient was bleeding from an ulcer, and the other was bleeding from hemorrhoids. One patient in the rivaroxaban group had major bleeding from a duodenal ulcer. No blood transfusions were performed in either group. In the aspirin group, three patients (1.52%) had a wound hematoma, two (1.01%) of whom needed aspiration while the other did not (minor bleeding). In the rivaroxaban group, there were three (1.56%) wound hematomas with aspiration and two (1.04%) gingival bleedings (minor bleeding). A combination of major bleeding and clinically relevant bleeding occurred in five patients (2.5%) in the aspirin group and in six (3.1%) patients in the rivaroxaban group. The incidence of any bleeding event among the two groups indicated no significant differences (*P* = 0.77, Table 3). During the 90‐day follow‐up, no death, myocardial infarction, stroke, or surgical site infection occurred in the two groups.

**Table 3:** Comparisons of bleeding between the two groups

Outcomes	Aspirin (n = 198)	Rivaroxaban (n = 192)	*P‐*value
	*no.%*	*no.%*	
Major bleeding	2(1.0)	1(0.5)	1.00
Clinically related bleeding	2(1.0)	3(1.6)	0.68
Minor bleeding	1(1.5)	2(1.0)	0.62
Any bleeding	5(2.5)	6(3.1)	0.77

### *Subgroup Analyses*

According to the fracture site, the patients were divided into two subgroups (femoral neck fracture group and intertrochanteric fracture group), and there was no significant difference in primary effectiveness and safety outcomes between the two groups (Table 4).

**Table 4:** Primary effectiveness and safety outcomes, according to fracture site

Outcomes	Femoral neck fracture			Intertrochanteric fracture of the femur		
	Aspirin	Rivaroxaban		Aspirin	Rivaroxaban	
	n = 72	n = 69	*P‐*value	n = 126	n = 123	*P*‐value
	no.(%)			no.(%)		
Venous thromboembolism	6(8.33)	4(5.8)	0.745	7(5.56)	7(5.56)	1.00
Pulmonary embolism	0	0	‐	1(0.79)	0	1.00
Major bleeding	1(1.39)	1(1.45)	1.00	1(0.79)	0	1.00
Clinically relevant bleeding	1(1.39)	1(1.45)	1.00	1(0.79)	2(1.63)	0.61
Minor bleeding	1(1.39)	1(1.45)	1.00	0	1(0.81)	0.49
Any bleeding	3(4.17)	3(4.35)	1.00	2(1.59)	3(2.44)	0.68

## Discussion

Extended prophylaxis with LMWH is inconvenient, has low patient compliance, and increases the cost to patients who have undergone THA, TKA, and HFS^48^, ^49^, ^50^, ^51^. The rivaroxaban was not suitable for patients with renal insufficiency, low‐income patients, and patients with a high risk of bleeding^32^, ^34^, ^37^. The European guidelines on perioperative VTE prevention recommend use of aspirin for hip fracture surgery (Grade 1C), but adequate large‐scale trials with proper study designs should be conducted^39^. We compared the efficacy and safety of aspirin and rivaroxaban sequential enoxaparin for VTE prevention after HFS. We found that the incidence of VTE and any bleeding in aspirin was equivalent to the direct oral anticoagulant rivaroxaban for extended prevention of VTE.

There were some limitations in our trial. Since the recruitment of patients frequently occurred postoperatively, the trial population did not consist of a whole inception cohort treated in accordance with a standardized strategy. Thus, we could not calculate the absolute event rates of thromboembolic or bleeding complications associated with each of the two prophylaxis protocols. Due to different races, the incidence of deep venous thrombosis after major orthopaedic surgery in China is higher than that reported abroad, but the rate of PE is close to zero^52^. Sample size was estimated based on the incidence of deep venous thrombosis after the intervention of rivaroxaban, which was on the basis of a survey of Chinese thromboembolism experts, orthopaedic surgeons, and literature^47^. A minimal, clinically important difference was established to be 13 percentage point on the basis of literature^32^. Our sample size is lower than that of Anderson's two studies, which used non‐inferior test^15^, ^40^, and our research uses equivalence test. To account for withdrawal of consent or loss to follow‐up over the course of the trial, we increased the final sample size by 25.8% to 390, but no statistical difference had been found, which indicates that the efficacy and safety of aspirin and rivaroxaban was equivalent. In our study, the surgery waiting time was more than 4 days, which was longer than the time stipulated in the guidelines for hip fracture in the elderly^27^, ^53^, ^54^, ^55^. Additionally, the patients with hip fracture were mostly more than 65 years old, accompanied by hypertension, diabetes, bronchitis, coronary heart disease, and other diseases. Nonsurgical treatment may increase complications, such as respiratory and urinary system infection and venous thrombosis. Therefore, surgical treatment is the preferred treatment, and surgery should be performed early for patients who can tolerate it according to the guidelines for hip fracture^56^. Kostuj *et al*.^57^ found that unnecessary examination before surgery may lead to delayed surgery, while surgery waiting time of more than 2 days would increase the mortality rate. Adequate preoperative evaluation is required for elderly hip fracture patients, but surgery is often delayed. Librero *et al*.^58^ assessed the prognosis of 56,500 patients with hip fractures over 60 years old through cohort studies, suggesting that the timing of surgery is not an independent risk factor for short‐term mortality after controlling for age, gender, complications, and other confounding factors. Two studies on prognostic factors for mortality after hip fracture showed that age, sex, and number of comorbidities influenced both early and late mortality in patients suffering from proximal hip fractures, and the option of operating within 3 days was not a valid alternative^41^, ^42^. In our study, the patients in the two groups had a surgery waiting time of more than 4 days, and they were fully evaluated before and after surgery and were administered mechanical prophylaxis before surgery and extended prevention of VTE after surgery according to guidelines^27^, ^59^. No deaths caused by thrombosis occurred in the 3 months after surgery.

LMWH is effective for extended anticoagulation after HFS, but it is associated with the risk of major bleeding. A multicenter, randomized double‐blinded trial assessed 656 patients who underwent HFS and received fondaparinux to prevent VTE for 6 to 8 days, followed by randomization to receive fondaparinux or placebo for 19 to 23 days. The rates of DVT diagnosed on venography were 1.4% *versus* 35%, demonstrating a trend toward more major bleeding events in the fondaparinux group.^18^ Fisher *et al*.^19^ studied 469 patients who underwent HFS and received semuloparin to prevent VTE for 7 to 10 days, followed by randomization to receive semuloparin or placebo for 19 to 23 days. The rates of DVT diagnosed on venography were 3.9% vs 17% for semuloparin and placebo, respectively, and they were accompanied by two patients with clinically relevant bleeding events in the semuloparin group (312 patients) and none in the placebo group. A randomized trial found that extended prophylaxis for 28 days with aspirin was noninferior to and as safe as dalteparin for the prevention of VTE after THA in patients who initially received dalteparin for 10 days^40^. The rates of symptomatic VTE were 0.3% (1/380) vs 1.3% (5/398). Clinically significant bleeding events occurred in five patients (1.3%) receiving dalteparin and two patients (0.5%) receiving aspirin. Anderson *et al*.^15^ performed a multicenter, double‐blind, randomized, and controlled trial to compare aspirin or rivaroxaban for VTE prophylaxis THA or TKA. They found that among patients who received 5 days of rivaroxaban prophylaxis and then continued rivaroxaban or aspirin for an additional 9 days after TKA or for 30 days after THA, there was no significant difference from rivaroxaban in the prevention of symptomatic VTE. The rates of VTE were 11 of 1707 patients (0.64%) in the aspirin group and 12 of 1717 patients (0.70%) in the rivaroxaban group. Major bleeding complications occurred in eight patients (0.47%) in the aspirin group and in five patients (0.29%) in the rivaroxaban group. Clinically important bleeding events occurred in 22 patients (1.29%) in the aspirin group and in 17 (0.99%) in the rivaroxaban group. In our study, the incidences of VTE were 6.6% (13/198) vs 5.7% (11/192) in the aspirin and rivaroxaban group (*P* = 0.83), respectively, with no lethal PE in either group. In our study, the higher incidence of VTE (aspirin group: 6.6%, rivaroxaban group: 5.7%) than in the above trials may be due to the following reasons. First, the anticoagulants used in the extended prophylaxis were different. We used oral anticoagulation (aspirin and rivaroxaban) in our trial, whereas LMWH was prescribed in Eriksson's^18^ and Fisher's^19^ trials. Second, the studied diseases were different. Anderson *et al*. used aspirin in their two RCT trials for extended prophylaxis after total hip or knee arthroplasty^15^, ^40^. Third, the criteria used were different. Anderson *et al*. performed ultrasound or venography only in patients with symptoms or suspected thrombosis or PE, and asymptomatic thrombosis was not evaluated^15^, ^40^. Because the patients in our study had some combined diseases and the waiting time for surgery was long, the length of hospitalization was prolonged. Ultrasonography of the lower extremity vein was a routine examination before and after the operation in our study and led to an increased incidence of VTE.

Major bleeding events in the aspirin and rivaroxaban groups occurred in two patients (1.0%) and one patient (0.5%) (*P* = 1.0). This incidence is higher than in Anderson's^15^ trial (major bleeding: 0.47%; any bleeding: 1.29%). The reasons are as follows: the number of patients in our trial was small. Aspirin monotherapy is not recommended as an appropriate pharmacological prophylaxis for patients after HFS (Grade D)^27^. In our trial, enoxaparin was used for anticoagulation 5 days after HFS, followed by aspirin for the remaining 16 days. It was difficult to determine whether bleeding was predominantly related to the initial postoperative enoxaparin, to the trial anticoagulant, or to a combination of both. These factors could potentially result in an overestimation of the rate of aspirin‐related bleeding. Thus, the rate of major bleeding events was higher in our trial than in those using LMWH as the sole anticoagulant for extended prophylaxis^18^, ^19^. In the prevention of VTE, LMWH can be adjusted according to weight, which can reduce postoperative bleeding complications. Although the incidence of VTE was higher, there were no fatal pulmonary embolisms in our study, which is consistent with Fisher's^19^ and Eriksson's^18^ trials and better than Anderson's^15^ results (one fatal PE). Our extended prophylaxis anticoagulation scheme was effective and safe.

The 2009 SIGN guidelines recommend that for patients undergoing HFS, LMWH may be used for pharmacological thromboprophylaxis (Grade A) and extended for 28 days starting 6 h after surgery (Grade A). The duration of anticoagulation was 21 days, 14 days, and 21 days respectively, in the studies of Powers *et al*.^60^, Gent *et al*.^61^, and Eriksson *et al*.^18^ examining the postoperative anticoagulation of hip fracture, with the incidence of VTE of 40.9%, 27.8%, and 1.4%, respectively. The incidence of VTE in Eriksson's trial was the lowest. In our study, the incidence of VTE, PE, and any bleeding events were lower with the extended prophylaxis for 21 days, which proved that our treatment program was reasonable.

Despite some limitations, our findings are clinically important. Our study shows that aspirin and rivaroxaban was equally effective and safe for extended treatment after HFS. We chose clinically relevant and important endpoints for the primary effectiveness analysis. In our trial, all patients were examined for lower limb venous ultrasound before and after surgery to confirm the presence of VTE. Suspected PE requires further computed tomographic pulmonary angiography. All patients were followed up for 90 days after surgery.

### *Conclusions*

In summary, aspirin was equivalent to the direct oral anticoagulant rivaroxaban for prevention of VTE after HFS and an initial 5‐day postoperative course of enoxaparin. We found that aspirin may be an effective, safe, convenient, and cheap alternative for extended prophylaxis after HFS. We recommend future large‐sample, prospective, double‐blind, randomized controlled trials to evaluate the efficacy and safety of aspirin for extended prophylaxis after hip fracture.

## Declarations

### *Ethics Approval and Consent to Participate*

The present study was approved by the Medical Ethics Committee of Chengdu Fifth People's Hospital. At the beginning of the study, all subjects were informed of the purpose, the potential benefits, and the risks of the trial. All subjects were voluntary participants who provided informed consent.

### *Consent to Publish*

Not applicable.

### *Availability of Data and Materials*

The datasets used and analyzed during the current study available are from the corresponding author upon reasonable request.

### *Competing Interests*

The authors declare that they have no competing interests.

### *Authors' Contributions*

Q H collected the data, performed the measurements and analysis, participated in the study design, and drafted the manuscript. S‐X X and Z‐k Z participated in the study design, supervised the analysis and helped to draft the manuscript. Y Z and H‐B S collected the data and performed the measurement. B S designed the study, supervised the whole study process, and helped to draft and review the manuscript. All authors read and approved the final manuscript.
